# Glutamate Exacerbates Traumatic Brain Injury‐Induced Acute Lung Injury Through NMDAR/ROS/Ca^2+^ Signaling Pathway in Pulmonary Endothelial Cells

**DOI:** 10.1002/kjm2.70087

**Published:** 2025-09-10

**Authors:** Song Zhou, Ying‐Ying Lou, Xue‐Zhen Ying

**Affiliations:** ^1^ Department of Cardiothoracic Surgery Ningbo Medical Center Lihuili Hospital Ningbo Zhejiang Province China; ^2^ Department of Pain Ningbo Medical Center Lihuili Hospital Ningbo Zhejiang Province China; ^3^ Department of Pharmacy Ningbo Medical Center Lihuili Hospital Ningbo Zhejiang Province China

**Keywords:** Ca^2+^, glutamate, NMDAR, ROS, TBI‐ALI

## Abstract

Traumatic brain injury (TBI) causes a high level of blood glutamate, which triggers host defense by activating oxidative stress and inflammation response. However, the concrete mechanism underlying its exacerbating effects on acute lung injury (ALI) severity remains unknown. In the present study, we aim to demonstrate the special role of N‐methyl‐D‐aspartate receptor (NMDAR) in regulating glutamate‐related inflammation signaling to facilitate the sustaining injury. After the interventions, blood glutamate concentration was measured using HPLC‐MS/MS. The level of pro‐inflammation cytokines, wet/dry weight ratio, protein concentration, and lung injury score were measured to examine the severity of lung damage. The oxidative status was evaluated by measuring the levels of reactive oxygen species (ROS), malondialdehyde (MDA), superoxide dismutase (SOD) activity, and intracellular Ca^2+^ concentration. Endothelial cell dysfunction was assessed through dye extravasation assay and quantification of p‐NFAT, p‐p65, ICAM‐1, and VCAM‐1 expression levels. Results showed that glutamate activated the NMDAR pathway, inducing endothelial barrier dysfunction via ROS/MDA elevation and SOD suppression. This cascade promoted the concentration of Ca^2+^, activating both nuclear factor of activated T cells (NFAT) and nuclear factor kappa‐B (NF‐κB) pathway. Glutamate administration exacerbated NMDAR activation, leading to persistent lung injury following TBI. Memantine‐mediated NMDAR blockade effectively attenuated this injury. Our findings indicate that blood glutamate elevation may trigger TBI‐associated acute lung injury (TBI‐ALI) through endothelial NMDAR/ROS/Ca^2+^ signaling.

## 1 Introduction

Traumatic brain injury (TBI) is a life‐threatening syndrome that can be complicated by serious secondary injuries of peripheral organs [1]. However, traumatic brain injury induced acute lung injury (TBI‐ALI) emerged as a clinically significant complication, demonstrating alarmingly high morbidity and mortality rates. Multiple mechanistic theories, including both neuro and noneuro responses, have been proposed to elucidate TBI‐ALI pathophysiology, among which inflammatory cascades is still the key pathogenesis for pulmonary damage [2, 3]. Up to now, the precise counterregulatory mechanisms underlying TBI‐induced pulmonary pathology remain incompletely characterized.

Glutamate serves as the principal excitatory neurotransmitter in the mammalian central nervous system (CNS), participating in brain development, learning, and memory functions [4]. Notably, glutamate receptors and transporters exhibit extensive extra‐neuronal distribution, with significant expression detected in peripheral organs including pulmonary, hepatic, renal, gastric, and immune tissues [5]. Emerging evidence implicates that the pro‐inflammatory role of blood glutamate in the pathology of TBI‐ALI suggests the close relationship between glutamate and lung injury [6]. The N‐methyl‐D‐aspartate receptor (NMDAR), a ligand‐gated calcium channel, mediates glutamate‐induced excitotoxicity primarily through pathological calcium influx [7]. Glutamatergic communication through NMDAR occurs outside the CNS as well [8, 9]. A number of studies have suggested that NMDAR activates excitotoxicity in lung tissues by inducing a high‐permeability pulmonary edema and airway inflammation [10, 11]. For example, the noncompetitive NMDAR antagonist MK‐801 attenuated sepsis‐induced oxidant lung injury, accounting for some forms of ALI caused by NMDAR [12]. However, the precise molecular pathways through which glutamatergic signaling modulates pulmonary inflammation and contributes to ALI progression remain to be fully elucidated, warranting systematic investigation.

This study systematically investigates the glutamate/NMDAR axis in pulmonary pathology through integrated in vivo and in vitro approaches. Utilizing a well‐established TBI‐ALI murine model and glutamate‐stimulated cellular systems, we employed both pharmacological activation and inhibition of NMDAR to elucidate the sources of glutamate excess and delineate its mechanistic contributions to ALI pathogenesis.

## 2 Materials and Methods

### 2.1 Animal Experiments

Forty adult male C57BL/6 mice (8–10 weeks old and weight 20–22 g) were purchased from Shanghai laboratory and were randomly divided into five groups (*n* = 8/group) via Research Randomizer: Sham group, TBI group, Glutamate group (Glu), TBI+Glutamate (TBI+Glu) group, and TBI+Memantine (TBI+Me) group. Surgeons were blinded to drug interventions, while outcome assessors were blinded to group assignments. To establish the TBI model, a validated controlled cortical impact was performed as previously reported. After intraperitoneal injection of 1.5% pentobarbital sodium, the mouse was placed in a stereotaxic frame and underwent a craniotomy with a diameter of 3.5 mm over the left parietal cortex at the center between the bregma and the lambdoid suture. Mice from the TBI group, TBI+Glu group, and TBI+Me group were subjected to a strike on the dura mater at a 2 mm depth and 3.5 m/s rate for 100 ms by the electron cortical contusion impactor (PSI, Fairfax Station, VA) [13]. Then, the bone flap was repositioned and the surgical area was sutured closed. Sham animals underwent a craniotomy but without the impact. Glutamate (10 mM, 500 μL/1 mL, Solarbio, China) was given intraperitoneally 30 min after TBI, and memantine (5 mg/kg, Sigma, USA) was given 1 h before TBI, respectively. This reproducible TBI model was confirmed by the mortality and neurological deficit scores at 24 h post‐injury [14]. Exclusion criteria: Intraoperative dural rupture (*n* = 1), postoperative mortality within 24 h (*n* = 2). The surgical criteria inclusion: Successful TBI model establishment (post‐impact neurological score ≥ 8), exclusion: Positioning deviation > 0.5 mm (*n* = 0). The contusion volume was examined by TTC staining. Experimental protocols were approved by the animal research ethics committee of Shanghai Yinxi Biotechnology Company (No. 20200801).

### 2.2 Histological Analysis

Lung specimens were collected 24 h after the TBI model and fixed in 4% paraformaldehyde (PFA) at 4°C for 24 h. Then the tissues were embedded in paraffin, cut into sections (5‐μm), and stained with hematoxylin and eosin. Lung sections were scanned and evaluated in a blinded manner using a light microscope (Leica, Germany) at 200× magnification, and lung injury scores were determined by four independent parameters: alveolar edema, hemorrhage, the infiltration of inflammatory cells, and thickened alveolar septum [15].

### 2.3 Co‐Location Experiments

Take mouse lung tissue fixed with 4% paraformaldehyde and prepare paraffin sections. After antigen retrieval, incubate sequentially with anti‐CD31 (endothelial marker) and anti‐TNF‐α primary antibodies at 4°C overnight. Wash with PBS, then add corresponding fluorescently labeled secondary antibodies and incubate at room temperature in the dark for 1 h. Counterstain the nuclei with DAPI for 5 min, mount the slides, and capture images using a confocal microscope. Use ImageJ software to calculate the Pearson correlation coefficient (PCC) to quantify the degree of colocalization.

### 2.4 Detection of Tissue Wet‐to‐Dry Weight Ratio

Twenty‐four hours post‐TBI induction or sham procedure, tissues from the right upper lobe of the lung were surgically excised and weighed. Then lung tissues were subsequently dehydrated in an oven at 60°C for 48 h until constant mass was achieved. The wet‐to‐dry (W/D) weight ratio was calculated accordingly to measure the pulmonary edema severity.

### 2.5 Detection of Total Protein Concentration in Bronchoalveolar Lavage Fluid

Bronchoalveolar lavage fluid (BALF) was collected via tracheostomy. Pulmonary lavage was performed using three sequential 1‐mL aliquots of ice‐cold phosphate‐buffered saline (PBS), with a consistent fluid recovery rate of approximately 90%. The collected fluid was centrifuged (400 × *g*, 10 min, 4°C) and the supernatant was collected for subsequent protein quantification assays.

### 2.6 Evans Blue Stain

Pulmonary microvascular permeability was quantitatively assessed using the Evans blue extravasation technique. At 8 h post‐TBI, the mice received an intravenous tail vein injection of 1% Evans blue dye (20 mg/kg body weight). Following euthanasia, the left lung was perfused with normal saline to remove intravascular dye, blotted dry, and precisely dissected. Accurately weighing the lung tissue to 100 mg, 1 mL formamide was added to cover the lung tissue and placed in a 60°C water bath for 24 h. After removing the tissues and centrifuging them, the absorbance at 620 nm was detected by an enzyme‐labeled instrument (Sigma, USA).

### 2.7 Enzyme‐Linked Immunosorbent Assay Analysis for Cytokine Analysis

BALF was centrifuged (400 × *g*, 10 min, 4°C), and the supernatants were collected for Enzyme‐linked immunosorbent assay (ELISA) of the pro‐inflammatory cytokines (TNF‐α, IL‐1β, and IL‐6) as previously described [16]. Absorbances were recorded using a multifunctional microplate reader (Thermo Fisher Scientific, Waltham, MA, USA), with appropriate blank and standard curve controls included in each assay.

### 2.8 Flow Cytometry

Freshly isolated lung tissues were cleaned by ice‐cold PBS and cut into approximately 1 mm^3^ fragments. Tissue digestion was performed by incubating the fragments with 1 mL of 0.25% trypsase (w/v) in a 37°C water bath for 60 min, with periodic mechanical dissociation using a sterile pipette to ensure complete tissue dispersion. The cells were filtered by a 200 μm cell filter and washed twice with cold PBS. 2′,7′‐Dichlorodihydrofluorescein diacetate (DCFH‐DA; Abcam, Cambridge, MA, USA) was added to the medium to reach a working concentration of 20 μM and incubated at 37°C for 30 min. The incubated cells were collected and then re‐suspended with PBS, and the FITC fluorescence detection conditions were used for detection on flow cytometry (Becton Dickinson, Franklin Lakes, NJ, USA).

### 2.9 Western Blotting

Total protein from left lung tissues was extracted using RIPA buffer containing a protease inhibitor and phosphatase inhibitors. Cytoplasmic and nuclear protein fractions were isolated using a subcellular fractionation kit (#HR0241, BioLabPrime, China), followed by lysis and centrifugation to collect the supernatant. Protein concentration was measured using a bicinchoninic acid (BCA) protein assay kit (Thermo Fisher Scientific, USA). After denaturation, protein (50 μg) was loaded per lane and separated by 10% sodium dodecyl sulfate‐polyacrylamide gel electrophoresis (SDS‐PAGE) and transferred to a 0.22 μM polyvinylidene difluoride (PVDF) membrane (Bio‐Rad, Hercules, CA, USA) for 1.5 h. The membranes were subsequently incubated with 5% bovine serum albumin (BSA) for 1 h at room temperature and incubated at 4°C overnight with the primary antibodies: p‐NFAT (1:1000, PA5‐11456, Invitrogen), p‐p65 (1:2000, ab86299, Abcam), ICAM‐1 (1:1000, ab2213, Abcam), VCAM‐1 (1:1000, ab134047, Abcam), GluN1 (1:1000, ab109182, Abcam), and ACTIN (1:5000, ab8227, Abcam). Then the membranes were incubated with the horseradish peroxidase (HRP)‐conjugated secondary antibody at room temperature for 2 h. Protein bands were scanned using a scanner (Bio‐Rad), and image densities were normalized to ACTIN.

### 2.10 HPLC‐MS/MS Method

Serial blood samples were obtained through a tail cut at 2, 6, and 24 h post‐TBI, then collected into anticoagulant tubes. After centrifugation (12,000 × *g*, 10 min, 4°C), the supernatant was collected for HPLC‐MS/MS analysis. A Shimadzu Prominence ultra‐fast liquid chromatography (UFLC) system (Columbia, MD, USA) coupled to a 4000 QTRAP tandem mass spectrometer system (AB SCIEX, Concord, ON, CA) was used for this study. An XBridgeC18 column (2.1 × 50 mm, 5 μm; Waters, Milford, MA, USA) was used for separation at 40°C with a flow rate of 0.4 mL/min. The mobile phase consisted of (A) 0.1% formic acid in water and (B) methanol, with the following gradient program: 0–0.8 min, 5% B; 0.8–1.5 min, 5%–95% B; 1.5–2.0 min, 95% B; 2.0–3.0 min, 95%–5% B. Mass spectrometric detection was performed in positive ion mode using multiple reaction monitoring (MRM) with the following transitions: glutamate m/z 148.0 → 84.0 and orinase m/z 271.4 → 155.0. Optimal MS parameters were: ion source temperature 550°C, ion spray voltage −4500 V, curtain gas 35 psi, nebulizer gas (GS1) and heater gas (GS2) both at 55 psi. Compound‐specific parameters included declustering potential (DP) of 80 V and collision energy (CE) of 23 V for glutamate, and DP 110 V with CE 23 V for orinase.

### 2.11 Ca^2+^ Concentration, MDA, and SOD Measurement

The Ca^2+^ concentration was measured by a p commercial calcium assay kit (Nanjing Jiancheng Bioengineering Institute, China). At 24 h after the TBI model and sham group, the right lower lung was removed and disposed of with a grinder. Then the lung tissue was centrifuged at 12,000*g* for 10 min at 4°C and the protein concentration of the supernatant was detected by a BCA protein assay kit (Thermo Fisher Scientific, USA). For calcium measurement, 10 μL supernatant was mixed with 10 μL calcium assay buffer and 250 μL methylthymol blue chromogenic reagent, followed by incubation at 37°C for 5 min. The optical density (OD) was detected by a Multiskan GO microplate reader (51119200, Thermo Fisher Scientific) with an excitation wavelength of 610 nm. Finally, the Ca^2+^ concentration was calculated by the OD value and the protein concentration of the supernatant. Parallel samples were processed for oxidative stress markers: malondialdehyde (MDA) content and superoxide dismutase (SOD) activity were determined using commercial kits (S0131S and S0087, respectively, Beyotime Biotechnology, China) following the manufacturer's instructions.

### 2.12 Cell Culture

Human pulmonary microvascular endothelial cells (HPMVECs) were purchased from Cell Applications Inc. (San Diego, CA, USA; Catalog No. 300K‐05a) and cultured in RPMI‐1640 medium (Thermo Fisher Scientific, Waltham, MA, USA) with 1% penicillin–streptomycin and 10% fetal bovine serum (FBS, Gbico, Grand Island, NY, USA) at 37°C in a humidified atmosphere containing 5% CO_2_.

### 2.13 Cell Counting Kit‐8 Assay

HPMVECs were seeded in 96‐well plates at a density of 1 × 10^4^ cells/well. After being placed in an incubator for 24 h, glutamate (Sigma‐Aldrich, St. Louis, MO, USA) was added to the wells for the next 24 h. Subsequently, 10 μL of Cell Counting Kit‐8 (CCK‐8) reagent (Beyotime, Biotechnology, Shanghai, China) was added to each well for 2 h, and the absorbance at 450 nm was measured using a Multiskan GO microplate reader (Thermo Fisher Scientific, Waltham, MA, USA).

### 2.14 Immunofluorescent Microscopy

HPMVECs were grown on coverslips in 6‐well plates (Corning, NY, USA) overnight and followed by stimulation with glutamate for 12 h. After washing with PBS twice, they were fixed in 4% paraformaldehyde and then permeabilized with 0.3% Triton X‐100 at 4°C. A 5% donkey serum was used to block the nonspecific binding sites at room temperature for 1 h. The cells were incubated with primary antibodies against p‐NFAT (1:200; ab2722, Abcam) and p‐NF‐κB (1:100; #3033, Cell Signaling Technology) overnight at 4°C. After washing with PBS three times, the cells were incubated with fluorescent secondary antibodies (Jackson, USA) dylight 488 goat anti‐rat IgG (H + L) and dylight 594 donkey anti‐rabbit IgG (H + L) for an hour at room temperature. Nuclei were counterstained with DAPI (1 μg/mL; D9542, Sigma‐Aldrich), and fluorescent images were acquired using a Leica DMi8 inverted microscope (Leica Microsystems).

### 2.15 Statistical Analysis

All statistical analyses were conducted using GraphPad Prism 9.5 (GraphPad Software Inc., San Diego, CA, USA). For multiple group comparisons, one‐way analysis of variance (ANOVA) was performed, followed by post hoc Student–Newman–Keuls (SNK) test for pairwise comparisons when ANOVA indicated significant differences (*F* ratio *p* < 0.05). *p*‐value < 0.05 was considered statistically significant. Data are presented as mean ± standard deviation (SD) from at least three independent experiments.

## 3 Results

### 3.1 Blood Glutamate Level Was Increased After TBI‐ALI

**FIGURE 1:**
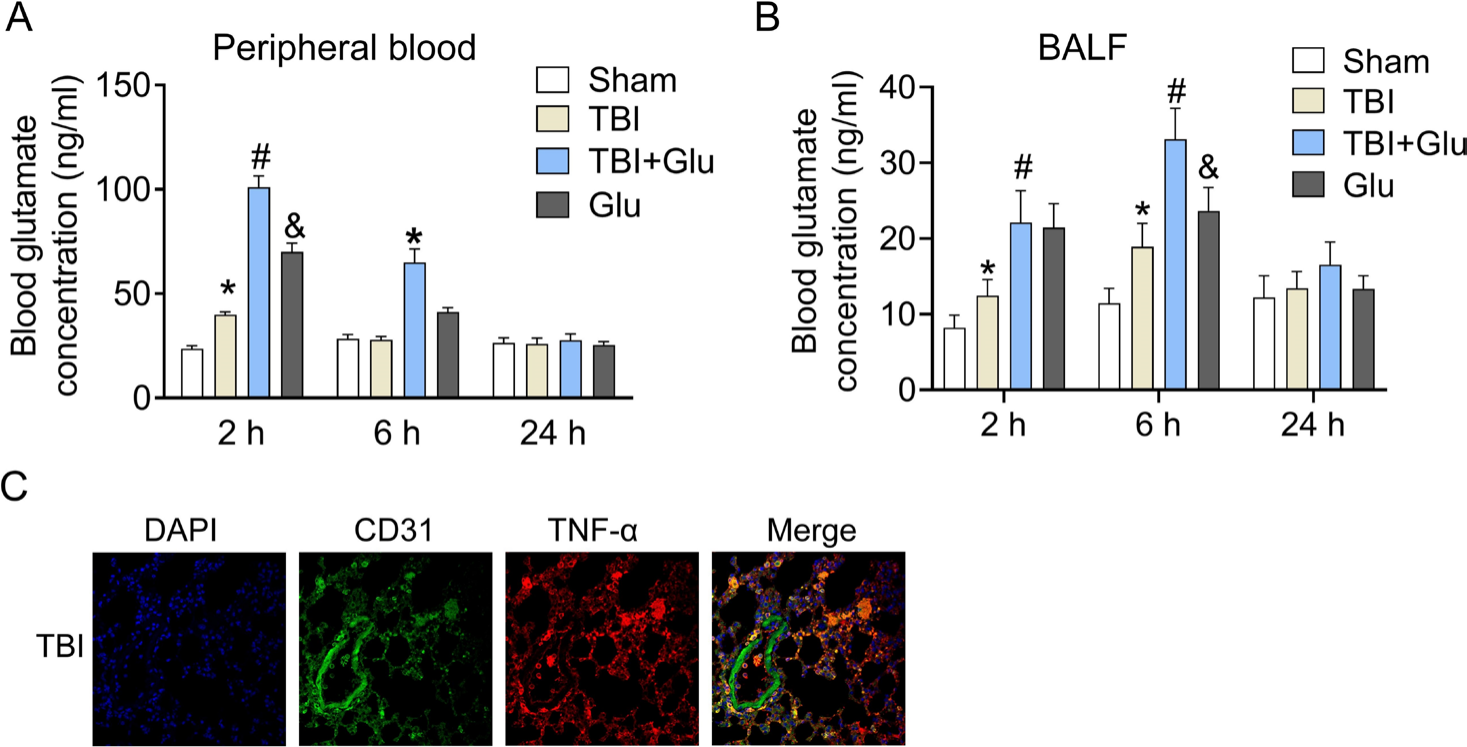
Blood glutamate concentration increased after TBI. (A) The level of blood glutamate concentration was detected at 2, 6, and 24 h. (B) The glutamate level in BALF was also determined by ELISA at 2, 6, and 24 h. (C) The co‐location experiment examined the co‐expression of CD31 and TNF‐α. Results represent the mean ± SEM of independent experiments of animals (*n* = 8). *p < 0.05 versus Sham group; ^#^ *p* < 0.05 versus TBI group; ^&^ *p* < 0.05 versus TBI + Glu group.

To explore whether glutamate is the main source of TBI‐ALI, we detected the level of blood glutamate after TBI‐ALI. The results showed that the blood glutamate concentration in the TBI group modestly and transiently altered at 2 h and turned back to normal level at 6 h and the next 24 h compared with the Sham group. However, injection of glutamate intraperitoneally in the TBI + Glu group and Glu group induced higher blood glutamate concentration at 2 h, which declined for the next 6 h and fell to regular level at 24 h (Figure 1A). The results indicated that blood glutamate is involved in the development of TBI‐ALI. The glutamate level in BALF was also determined by ELISA. As shown in Figure 1B, the glutamate concentration in the TBI group was increased at 2 and 6 h after TBI‐ALI compared with the Sham group. The injection of glutamate intraperitoneally in the TBI + Glu group and Glu group induced higher blood glutamate concentration at 2 and 6 h, which declined and fell to regular level at 24 h. Moreover, we performed a co‐location experiment to examine the co‐expression of CD31 and TNF‐α. The results were shown in Figure 1C; the PCC value was 0.781 ± 0.13. This indicated that CD31 and TNF‐α were co‐expressed in the lung tissue in TBI mice.

### 3.2 NMDAR Aggravated the Injury of TBI‐ALI

**FIGURE 2:**
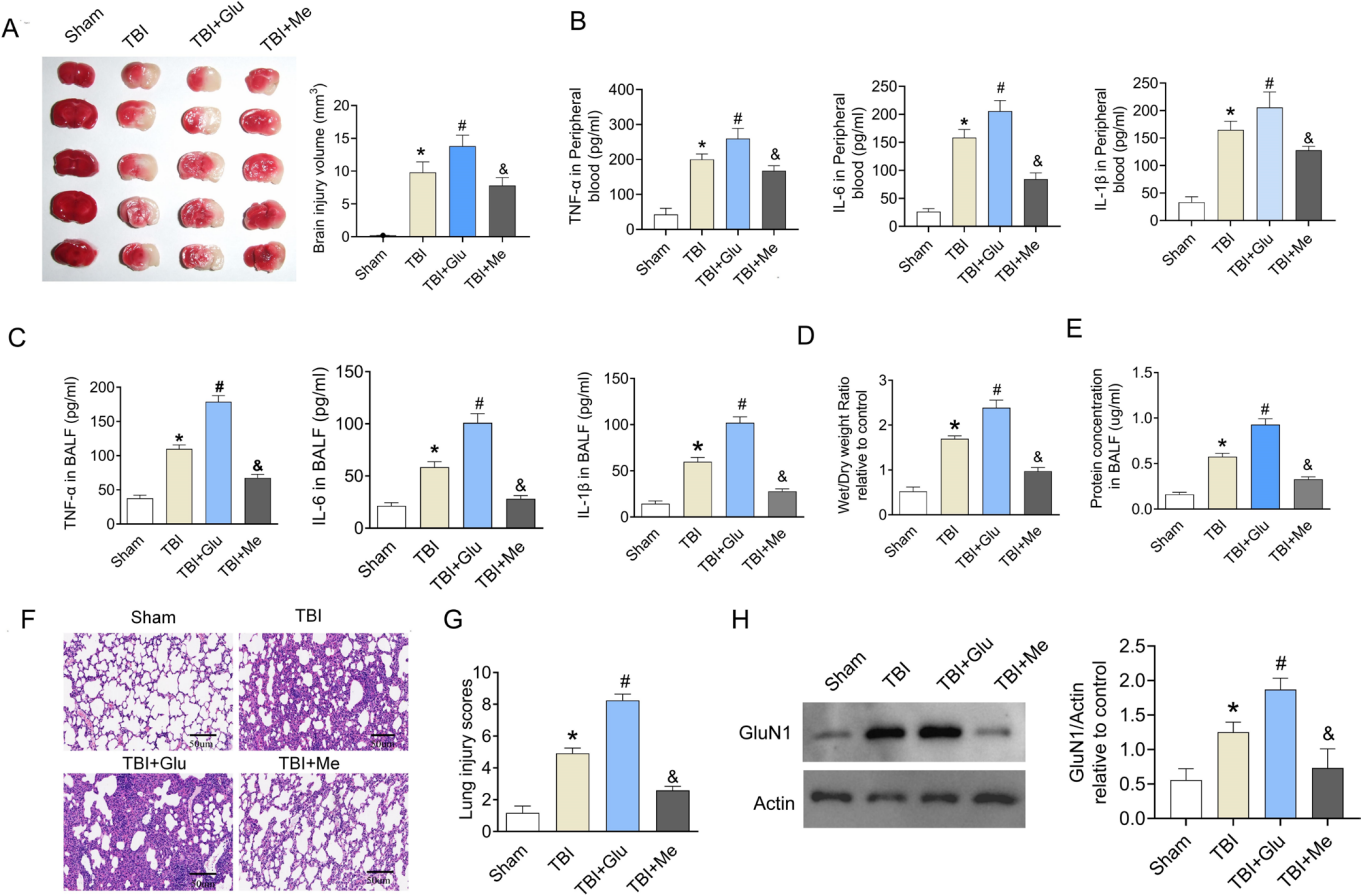
Changes of serious lung injury after TBI‐ALI. (A) The TTC staining was performed to evaluate the brain injury volume. (B) The level of cytokines (TNF‐α, IL‐1β, and IL‐6) in peripheral blood. (C) The level of cytokines (TNF‐α, IL‐1β, and IL‐6) in BALF. (D, E) The wet/dry weight ratio and the protein concentration are detected. (F, G) Corresponding lung H&E staining and acute lung injury scores. (H) The expression of GluN1 in HPMVECs. Scale bar, 200 μm. Results represent the mean ± SEM of independent experiments of animals (*n* = 8). *p < 0.05 versus Sham group; ^#^ *p* < 0.05 versus TBI group.

To assess NMDAR's role in TBI‐ALI, we pharmacologically modulated NMDAR signaling using glutamate (agonist) and memantine (antagonist) in a TBI model, quantifying effects on lung injury parameters. The TTC staining was performed to evaluate the brain injury volume. As shown in Figure 2A, the brain injury volume was increased in the TBI group compared with the Sham group. Glutamate treatment promoted the brain injury volume, while memantine treatment decreased the brain injury volume. The injection of glutamate in the TBI + Glu group promoted the release of inflammation factors, while memantine decreased the level of TNF‐α, IL‐1β, and IL‐6. The level of inflammation factors (TNF‐α, IL‐1β, and IL‐6) in peripheral blood was detected. As shown in Figure 2B, the TNF‐α, IL‐1β, and IL‐6 levels were increased after TBI. The level of inflammation factors (TNF‐α, IL‐1β, and IL‐6) in BALF was detected. Compared with the Sham group, the level of pro‐inflammation cytokines including TNF‐α, IL‐1β, and IL‐6 in BALF was increased in the TBI group (Figure 2C). The injection of glutamate in the TBI + Glu group amplified those indicators furthermore when compared with the TBI group. Meanwhile, previous treatment with memantine decreased the level of TNF‐α and IL‐1β when compared with the TBI group, although the levels remained higher than the Sham group. For IL‐6, the concentration in the TBI + Me group was comparable to the Sham group. Besides, the level of wet/dry weight ratio (Figure 2D) and the protein concentration (Figure 2E) in BALF showed a similar tendency as the inflammation factors did. Histopathology (Figure 2F) and lung injury score (Figure 2G) showed a clear structure of the alveolar wall and no secretions in pulmonary interstitium in the Sham group, while manifested significant interstitial infiltration of inflammatory cells and thickening of the alveolar walls when challenged with TBI. The degree of lung injury was much more serious in the TBI + Glu group and reversed in the TBI + Me group. These findings demonstrated the critical role of NMDAR in inducing the injury of TBI‐ALI. Moreover, the protein expression of GluN1 in HPMVECs was determined. It was shown that the GluN1 (functionally essential components of NMDARs) expression was increased in the TBI group, while the injection of glutamate promoted the GluN1 expression in the TBI + Glu group (Figure 2H). The previous treatment with memantine decreased the NMDAR expression.

### 3.3 NMDAR Mediated ROS‐Driven Ca^2+^ Signaling in Lung Tissue

**FIGURE 3:**
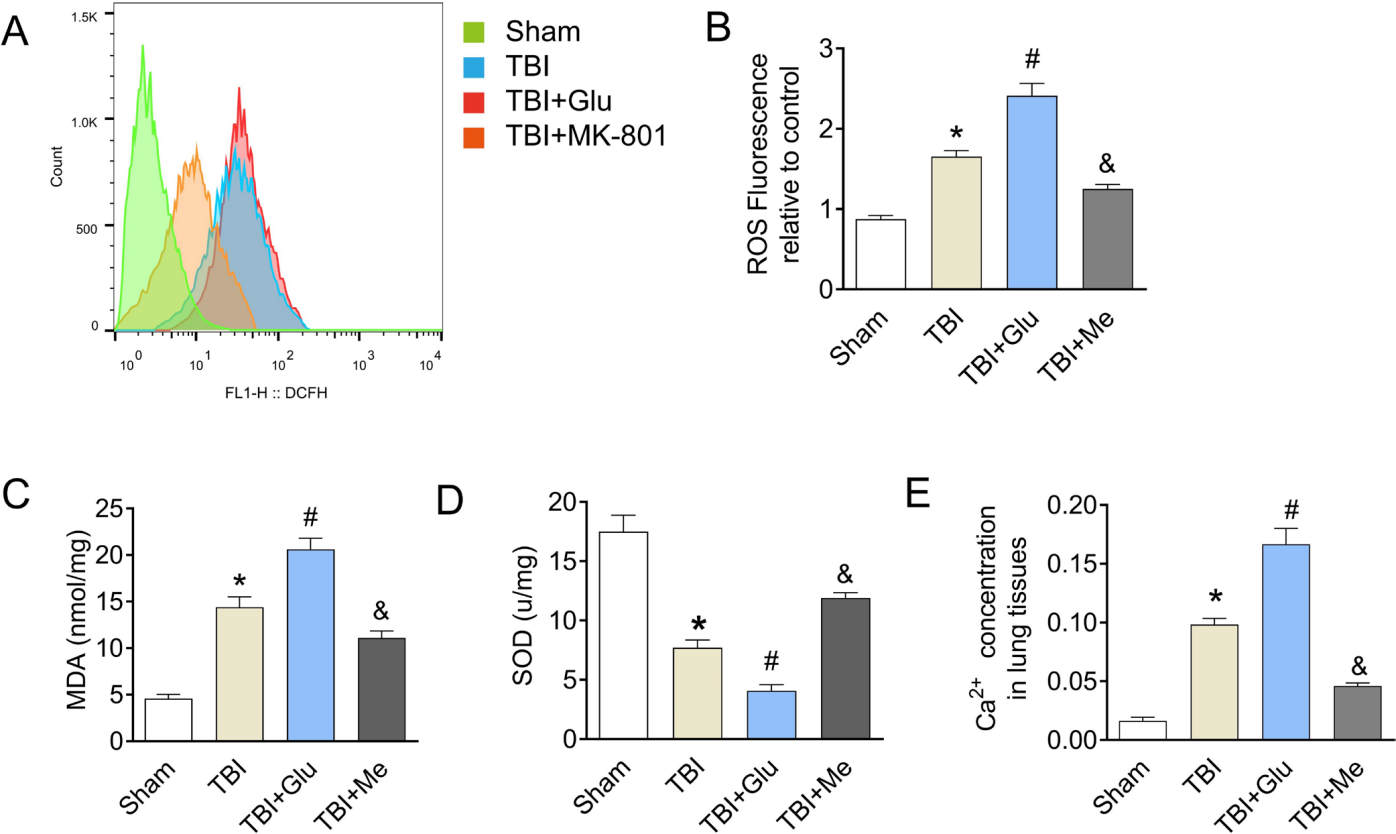
NMDAR activated oxidative indicator and Ca^2+^ concentration in lung tissues. (A, B) Representative flow cytometry images showing ROS production in the lung tissue in each group of mice. (C, D) Levels of the oxidative stress factors MDA and SOD between each group. (E) Comparisons of Ca^2+^ concentration are shown. Results represent the mean ± SEM of independent experiments of animals (*n* = 8). *p < 0.05 versus Sham group; ^#^ *p* < 0.05 versus TBI group.

We subsequently identified the levels of ROS, MDA, SOD, and Ca^2+^ in the lung tissue. The results demonstrated elevated ROS (Figure 3A,B) and MDA (Figure 3C) levels, along with reduced SOD activity, in the TBI group compared to the Sham group. The levels of ROS and MDA were higher in the TBI + Glu group and lower in the TBI + Me group than in the TBI group. The adverse result was observed in the levels of SOD (Figure 3D). Oxidative signaling and Ca^2+^ homeostasis are tightly linked to cellular processes in ALI. Based on the results above, we surmised that blood glutamate activated NMDAR in lung ROS production by regulating cytosolic Ca^2+^ levels. At 24 h after TBI, lung tissues were extracted for Ca^2+^ concentration measurement. As a result, the Ca^2+^ concentration increased significantly in the TBI group compared to the Sham group (Figure 3E). In addition, the injection of glutamate after TBI advanced the Ca^2+^ concentration superiorly when compared to the TBI group. The data in the TBI + Me group were lower than in the TBI group but still higher than in the Sham group, which showed the same consequence as the parameters in Section 3.2. The study showed that NMDAR‐controlled ROS‐driven Ca^2+^ influx was responsible for the severity of TBI‐ALI.

### 3.4 Endothelial Barrier Function via Ca^2+^ Induced Inflammation Response

**FIGURE 4:**
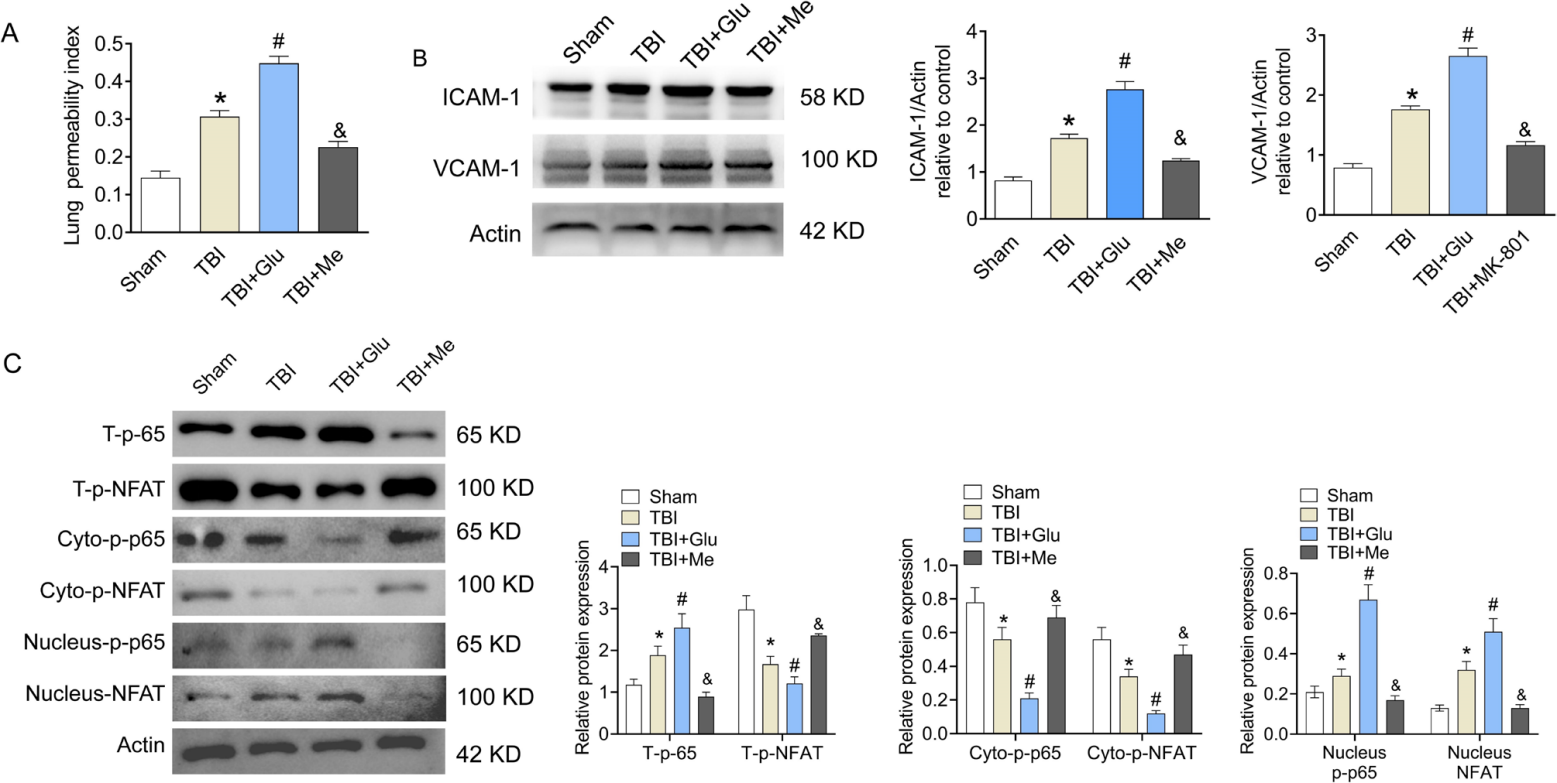
Endothelial cell damage and inflammation response. (A) Endothelial cell permeability was shown by Even Blue Stain. (B) The levels of ICAM‐1 and VCAM‐1 in lung tissues were detected by western blot. (C) The levels of p‐NFAT and p‐p65 in the cytoplasm and nucleus were tested by western blot. Results represent the mean ± SEM of independent experiments of animals (*n* = 8). *p < 0.05 versus Sham group; ^#^ *p* < 0.05 versus TBI group.

Endothelial cell permeability was shown by Even Blue Stain (Figure 4A). The data indicated that the endothelial cell permeability was increased in the TBI group, and Glu promoted the endothelial cell permeability. Me pretreatment inhibited the endothelial cell permeability. The levels of ICAM‐1 and VCAM‐1 in lung tissues were detected by western blot. As exhibited in Figure 4B, the expression of ICAM‐1 and VCAM‐1 in lung tissues was increased. Glu stimulated the expression of ICAM‐1 and VCAM‐1, while Me pretreatment inhibited the expression of ICAM‐1 and VCAM‐1. The levels of p‐NFAT and p‐p65 in the cytoplasm and nucleus were tested by western blot. Results represented that the T‐p‐p65 and Nucleus‐p‐p65 expressions were increased in the TBI group, while Glu promoted the T‐p‐p65 and Nucleus‐p‐p65 expressions. Meanwhile, the T‐p‐NFAT and Cyto‐p‐NFAT expressions were decreased in the TBI group, and Glu inhibited the T‐p‐NFAT and Cyto‐p‐NFAT expressions. Me showed the opposite effect on the expressions of p‐NFAT and p‐p65 in the cytoplasm and nucleus. These findings revealed that Ca^2+^‐dependent NFAT and NF‐κB activity may be the main reason for inflammation cytokine expression and subsequent vascular endothelial dysfunction.

### 3.5 Glutamate Alters NMDAR/ROS/Ca^2+^ Pathway in HPMVECs

**FIGURE 5:**
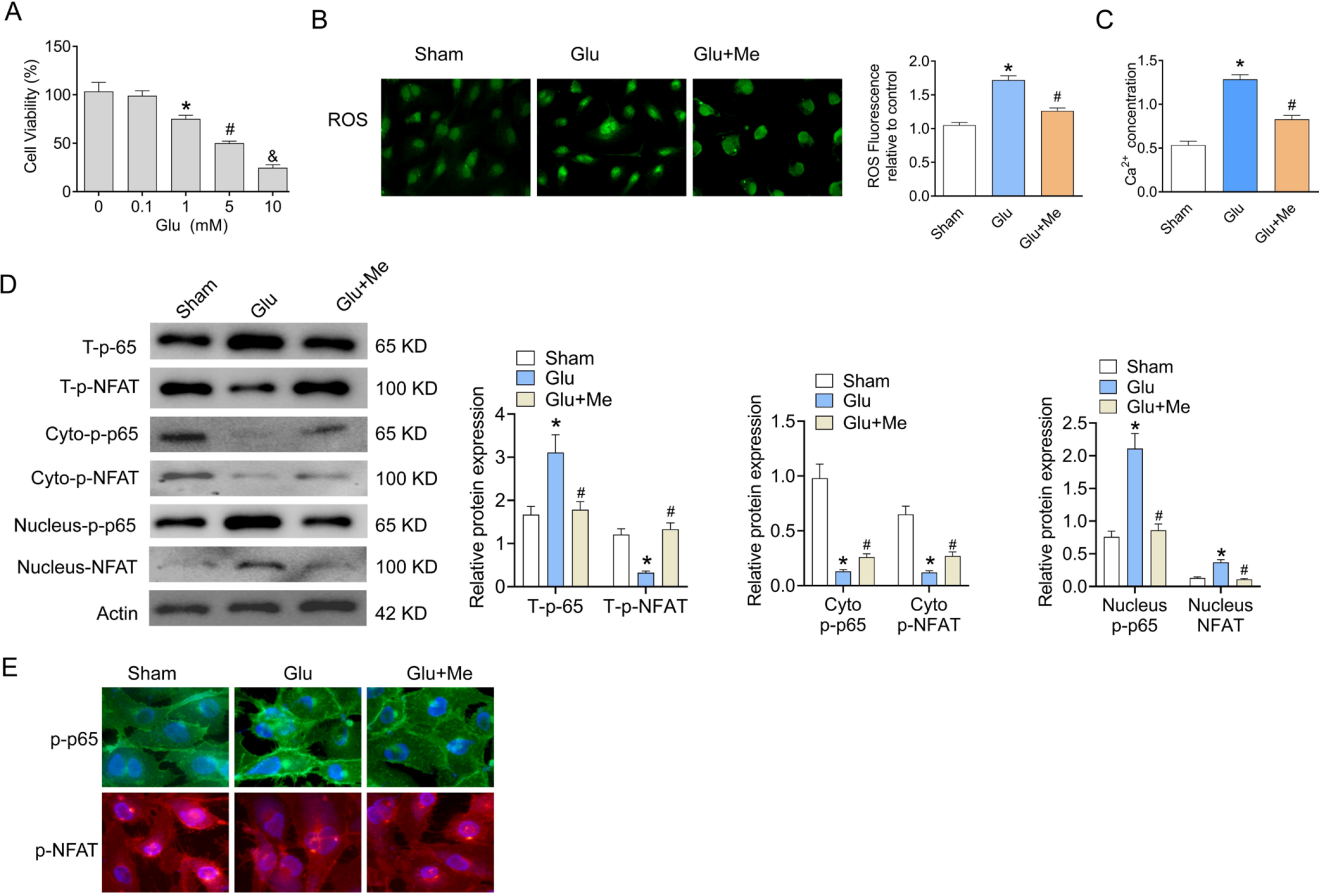
Glutamate alters NMDAR/ROS/Ca^2+^ pathway. (A) Cell viability (percentage of untreated control) of HPMVECs after the treatment of glutamate. (B) Immunofluorescence images showing ROS production in HPMVECs. (C) Comparison of Ca^2+^ concentration in each group. (D) The levels of p‐NFAT and p‐p65 in cytoplasm and nucleus were tested by western blot. (E) Immunofluorescence stain of p‐NFAT and p‐p65 in nucleus. Results represent the mean ± SEM of independent experiments of cells (*n* = 3). *p < 0.05 versus Sham group; ^#^ *p* < 0.05 versus Glu group.

In order to determine glutamate's role in the regulation of HPMVECs, the cytotoxic effect of glutamate is shown (Figure 5A). After exposure to 0.1, 1, 5, and 10 mM glutamate for 24 h, the relative cell viability of HPMVECs was measured. No cytotoxic effect of glutamate at a concentration of 0.1 mM was observed and chosen for further investigations. After the treatment of glutamate, a significant increase in ROS level detected by immunofluorescence was found in HPMVECs compared to the Sham group. However, the glutamate‐induced increase in ROS level was reversed by memantine (Figure 5B). Besides, the Ca^2+^ concentration was similar to the tendency of the ROS level (Figure 5C). The levels of p‐NFAT and p‐p65 in the cytoplasm and nucleus were tested by western blot. Results represented that the T‐p‐p65 and Nucleus‐p‐p65 expressions were increased in the Glu group, while Me reversed the T‐p‐p65 and Nucleus‐p‐p65 expressions. The T‐p‐NFAT and Cyto‐p‐NFAT expressions were decreased in the TBI group, and Me reversed the T‐p‐NFAT and Cyto‐p‐NFAT expressions. Immunofluorescence showed a significant increase in p‐p65 and reduction of p‐NFAT in nuclear accumulation in the Glu group compared to the sham group, while memantine reversed the result (Figure 5E). Generally, the manifestation of HPMVECs was similar to the result of lung tissues in TBI‐ALI, which meant that the glutamate‐induced NMDAR/ROS/Ca^2+^axis plays an important role in endothelial cells.

## 4 Discussion

**FIGURE 6:**
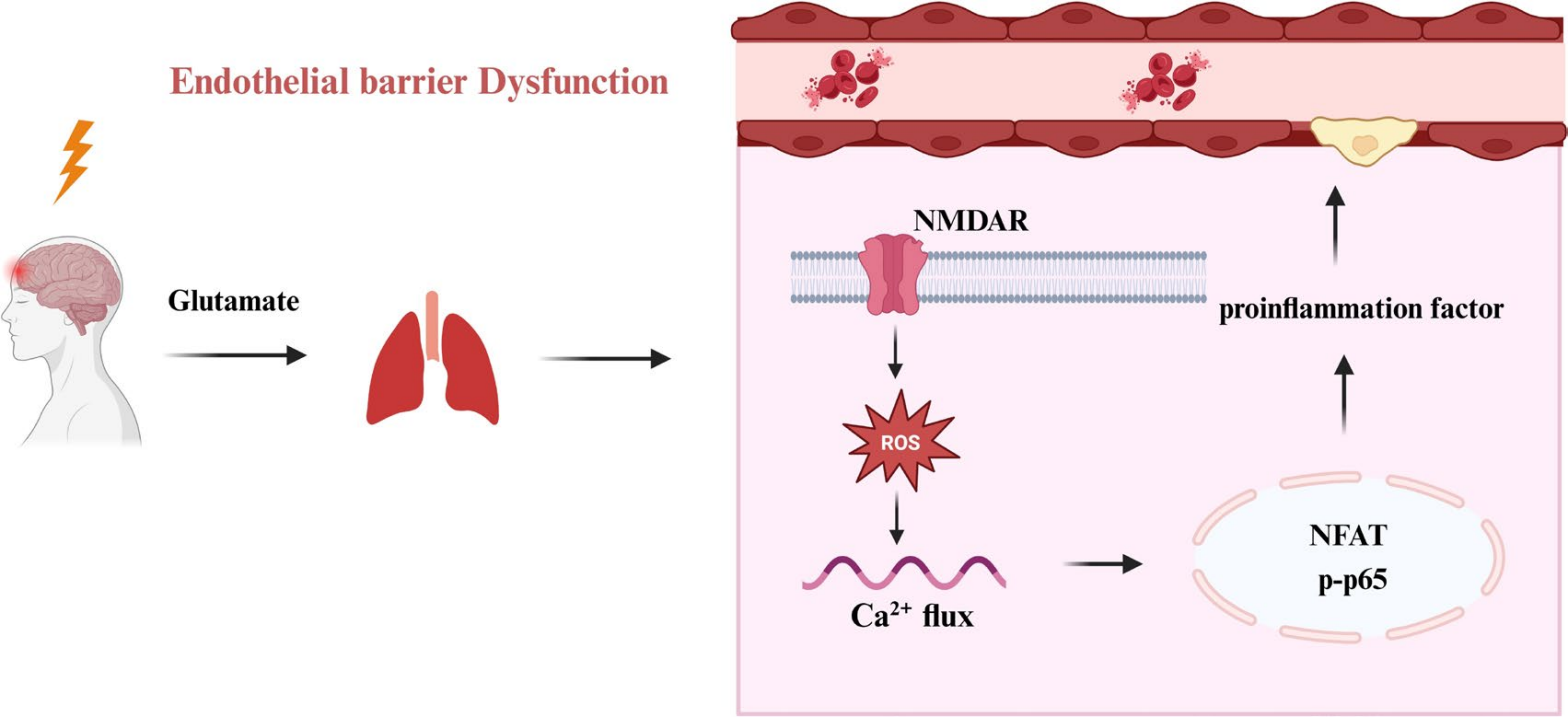
Schematic diagram of the potential mechanism involved in TBI‐ALI. TBI elevated the level of plasma glutamate, activating NMDAR‐ROS‐Ca^2+^ flow in endothelial cells, mediating the transcription factor NFAT and NF‐κB, which resulted in the augmentative inflammation response and the severity of ALI.

Our research demonstrates NMDAR serves as a critical regulatory checkpoint in amplifying glutamate‐related inflammation signaling in TBI‐ALI. We demonstrated that following the TBI model, increased blood glutamate activates the NMDAR/ROS/Ca^2+^ pathway in lung tissues, which promotes NFAT and NF‐κB mediated generation of pro‐inflammatory cytokines and promotes vascular barrier dysfunction, leading to the development of ALI (Figure 6).

Our study establishes N‐methyl‐D‐aspartate receptor (NMDAR) as a critical regulatory checkpoint in amplifying glutamate‐mediated inflammatory signaling during TBI‐ALI. Mechanistically, we demonstrate that elevated circulating glutamate levels following TBI trigger activation of the NMDAR/ROS/Ca^2+^ signaling cascade in pulmonary tissues. This pathway subsequently induces NFAT and NF‐κB‐dependent transcriptional upregulation of pro‐inflammatory cytokines while concurrently compromising vascular barrier integrity, ultimately culminating in ALI pathogenesis.

TBI‐ALI is one of the secondary injuries of TBI, which is a key factor influencing the prognosis of clinical patients [17]. Previous studies have primarily focused on the possible mechanisms for impairment of the pulmonary system, such as enhanced neuroinflammatory, increased apoptosis response, and excessive oxidative stress, which continue from hours to days in lung tissues [18, 19]. Among them, elevated blood glutamate induced excitotoxicity shows a critical role in the development of TBI‐ALI [6]. As a major excitatory neurotransmitter in the CNS, glutamate plays a well‐established role in neuro medicated signaling [20]. Glutamatergic function has also been implicated in a broad range area of lung injury, including inflammation, oxidative stress, and apoptosis [21]. In the CNS, glutamate binds to NMDAR and regulates neuronal oxidative stress in a Ca^2+^‐dependent manner [22]. Oxidative damage is a hallmark of ALI, which contributes to endothelial barrier function, leading to vascular tissue inflammation and damage [23]. Since glutamate is the main trigger of inflammation response of the lung, an urgent unanswered question is how glutamate modulates the development of ALI.

NMDAR is one of the glutamate receptors both in the central nervous system and peripheral tissues. Functional glutamatergic signaling and NMDAR expression have been reported in various pulmonary cells, including alveolar macrophages, alveolar type II cells, and neutrophils, leading to damage of lung tissues and airway [24, 25]. In consideration of the functional role of NMDAR in lung injury, we speculated that glutamatergic NMDAR modulated the development of TBI‐ALI. In the present study, we used glutamate as an agonist and memantine as an antagonist to explore the role of NMDAR. We measured the concentration of glutamate in blood at 2, 6, and 24 h after TBI. The results indicated that blood glutamate level increased at 2 h and declined to a normal level at the next 24 h after TBI. The injection of glutamate in the TBI + Glu group reached a higher level at 2 h with a slight drop at 6 h than the TBI group, returning to normal level at 24 h. In addition, we observed that the degree of cytokines, tissue‐edema level, and lung injury scores increased significantly in the TBI group than in the Sham group, which indicated a successful model establishment. Additionally, the injection of glutamate in the TBI + Glu group aggravated the severity of lung injury further when compared to the TBI group, while the injection of memantine before TBI reversed the change when compared to the TBI group, demonstrating that activated NMDAR may be the main resource for intensifying lung injury. The change of blood glutamate level after TBI is closely related to the serious degree of lung injury as NMDAR does, supporting the outcome that blood glutamate is the initiator for advancing the progress in TBI‐ALI. Overall, our data unequivocally demonstrate that the high concentration of glutamate in blood is a relative factor of prognosis for TBI‐ALI, which activates NMDAR to amplify ALI.

Though NMDAR has been reported to associate with the inflammatory response to lung injury, the evidence regarding how NMDAR regulates the pro‐inflammatory factor is not clear. Strong evidence exists that NMDAR, by promoting excessive ROS production and the entry of Ca^2+^ into cells, exhibits a crucial role in neuronal inflammatory damage associated with degenerative diseases such as strokes, epileptic seizures, and Alzheimer's disease [26]. Importantly, NMDAR exerted the pro‐inflammatory effect through inhibiting downstream Ca^2+^ signaling molecules in macrophages of the ALI model as well [27]. However, whether NMDAR could regulate the ROS/Ca^2+^ pathway in ALI is not clear. We showed that the oxidant index ROS and MDA were increased at 24 h after TBI, which reached a higher level in the TBI + Glu group. The injection of glutamate after TBI elevated ROS and MDA levels, while the injection of memantine before TBI lowered the level compared to the TBI group. At the same time, the anti‐oxidant indicator SOD produced a reversed result compared to ROS and MDA suggesting that activated NMDAR played an important role in ROS/Ca^2+^ regulation. The above results indicated that there is a close relationship between the NMDAR/ROS/Ca^2+^ pathway and the development of TBI‐ALI, which had never been covered in previous studies. Thus, the activated NMDAR resulting in a high Ca^2+^ concentration in lung tissues may explain the persistent increase in inflammatory signaling in TBI‐ALI.

Vascular endothelial dysfunction is an established pathological factor in multiple pathological conditions. It dynamically responds to extracellular environmental changes, exerting either beneficial or detrimental effects on the organism [23]. Hazardous substances such as endotoxins and viruses trigger innate immune cells to the vascular wall and activate the endothelium, producing abundant inflammatory cytokines and destroying the integrity of the endothelial barrier [28]. NFAT and the NF‐κB family are widely believed to play a critical roles in mediating the inflammatory response in endothelium, respectively [29, 30]. A rise in intracellular Ca^2+^ is an essential signal required for the inflammatory response, which is mediated by transcription factors, notably NF‐κB and NFAT [23, 31]. NFAT is basally phosphorylated and becomes activated when dephosphorylated by calcineurin [32]. Findings from our study showed that p‐NFAT and p‐p65, which present the phosphorylated state of NFAT and NF‐κB respectively, changed evidently after TBI. The expression of p‐NFAT decreased and p‐p65 increased adversely, indicating the transcriptional activity of NFAT and NF‐κB identically in the TBI group compared to the Sham group. Importantly, we found that the injection of glutamate after TBI even disappeared the expression of p‐NFAT and added to the expression of p‐p65 compared to the TBI group. On the contrary, the injection of memantine before TBI reduced the production of NFAT and NF‐κB compared to the TBI group. These results suggested that activated Ca^2+^ may promote cytokine generation through NFAT and NF‐κB dependently. However, it remains to be seen how NFAT cooperates with NF‐κB to modulate the cytokine secretion. Therefore, we measured VCAM‐1 and ICAM‐1, two well‐characterized vascular endothelial inflammatory markers, which elevated significantly after TBI compared to the Sham group, with a higher level after the injection of glutamate and a normal level in the injection of memantine. The results are consistent with the increase in NFAT and NF‐κB activity, indicating that NFAT and NF‐κB may be responsible for cytokine generation leading to the dysfunction of the endothelial wall. A similar result was shown in HPMVECs for glutamate treatment, demonstrating the excitability of glutamate in endothelial dysfunction. However, we have not conducted further research on locating the NMDAR on the endothelial cells and the relationship between ROS and inflammation, which limits the meaning of the results.

In summary, the present study has identified that NMDAR is a key mechanism in controlling glutamate signaling caused by TBI, thereby dampening inflammatory response in lung injury. We investigated that NMDAR, while being activated by elevated blood glutamate after TBI, facilitates the NFAT and NF‐κB‐driven sustained inflammatory injury in a ROS‐driven Ca^2+^ signaling, thus destroying the endothelial wall and accelerating the lung injury. Since this pivotal role of NMDAR, we speculate that NMDAR blockade might represent a promising therapeutic strategy for the treatment of TBI‐ALI.

## Conflicts of Interest

The authors declare no conflicts of interest.

## Data Availability

The data that support the findings of this study are available from the corresponding author upon reasonable request.
